# Collective multimode strong coupling in plasmonic nanocavities

**DOI:** 10.1515/nanoph-2024-0618

**Published:** 2025-03-21

**Authors:** Angus Crookes, Ben Yuen, Angela Demetriadou

**Affiliations:** School of Physics and Astronomy, 1724 University of Birmingham, B15 2TT Birmingham, UK

**Keywords:** quantum, plasmonics, multimode, strong coupling, nanocavities, cavity QED

## Abstract

Plasmonic nanocavities enable access to the quantum properties of matter but are often simplified to single mode models despite their complex multimode structure. Here, we show that off-resonant plasmonic modes in fact play a crucial role in strong coupling and determine the onset of a novel collective interaction. Our analysis reveals that *n* strongly coupled plasmonic modes introduce up to *n*(*n* + 1)/2 oscillation frequencies that depend on their coupling strengths and detuning’s from the quantum emitter. Furthermore, we identify three distinct regions as the coupling strength increases: (1) single mode, (2) multimode and (3) collective multimode strong coupling. Our findings enhance the understanding of quantum dynamics in realistic plasmonic environments and demonstrate their potential to achieve ultra-fast energy transfer in light-driven quantum technologies.

## 1 Introduction

Strong coupling between light and matter is essential across various fields, as it connects nanophotonics, materials science, chemistry and quantum technologies [1], [2], [3]. Strong light–matter coupling enables quantum emitters (QE) to coherently exchange energy with light, creating new polaritonic states that play a critical role in quantum information systems [4]. To reach the strong coupling regime, QEs (such as molecular dyes, cold atoms and quantum dots) must be placed within regions of intense light, where light–matter interaction rates outpace losses. Therefore, plasmonic nanocavities (such as metallic nanostructures, dimers and nanoparticle on mirror cavities) are ideal for realising strong coupling [5], [6], [7], [8], [9], [10], [11], [12], [13] as localised surface plasmons allow for exceptional subwavelength light confinement and extreme coupling strengths [14], [15]. In addition, they are straightforward to synthesise, chemically stable and facilitate the precise positioning and alignment of molecules for reliable and highly reproducible experiments [16], [17], [18].

Theoretical descriptions reveal how these new polariton states emerge and continue to guide experiments towards the generation of new quantum states. However, although significant advancements have been made in describing QEs within plasominc nanocavities – accounting for more complex vibrational structures and larger numbers of molecules [19], [20], [21] – usually the underlying assumption is that they interact with just a single cavity mode. The single mode approximation originates from the analysis of Fabry–Pérot and photonic crystal cavities [22], [23], [24], [25], where modes are spectrally separated relative to their coupling strengths. However, multimode coupling has been shown to play an important role experimentally in some optical cavities [26], [27], [28], [29]. Plasmonic nanocavities in particular support a dense collection of modes that overlap in frequency, and often all exhibit significant field enhancements [30], [31], [32]. Although coupling to multiple plasmonic modes has been previously considered [33], [34], [35], [36], [37], [38] in general, the underlying origin and impact of the complex quantum dynamics that emerge in these multimode systems is still not known.

In this work, we demonstrate that multiple, off-resonant plasmonic modes can significantly dominate the quantum dynamics of QEs in plasmonic nanocavities. In particular, we show that *n* strongly coupled modes introduce up to *n*(*n* + 1)/2 distinct oscillation frequencies in the QEs excited state population. These frequency components depend on the number of strongly coupled modes, and their respective coupling strengths and detunings from the QE. In fact, we identify three distinct regions defined by the dipole moment: (1) single mode strong coupling, (2) multimode strong coupling and (3) collective multimode strong coupling. Our results provide a comprehensive understanding of the quantum dynamics in multimode environments and demonstrate how realistic plasmonic nanocavities can be used to achieve ultra-fast energy transfer, for use in light-driven quantum technologies.

## 2 Strong coupling in multimode nanocavities

The interaction between multiple plasmonic modes and a single quantum emitter (QE) can be described effectively using an open quantum system formalism [38], [39]. In this form, the density operator *ρ*(*t*) evolves under:

(1) $$\dot{\rho }\left(t\right)=-i\left[H,\rho \right]+\overset{n}{\underset{\xi }{\sum }}\kappa_{\xi }\left(a_{\xi }\rho a_{\xi }^{†}-\frac{1}{2}\left\{a_{\xi }^{†}a_{\xi },\rho \right\}\right)$$

with Hamiltonian,

(2) $$H=\underset{H_{0}}{\underset{︸}{\overset{n}{\underset{\xi =1}{\sum }}\omega_{\xi }a_{\xi }^{†}a_{\xi }+\frac{\omega_{0}}{2}\sigma_{\text{z}}}}+\underset{H_{\text{int}}}{\underset{︸}{\overset{n}{\underset{\xi }{\sum }}g_{\xi }\left(r\right)a_{\xi }^{†}\sigma +g_{\xi }^{*}\left(r\right)a_{\xi }\sigma^{†}}}$$

where $a_{\xi }^{†}$ and *a*_*ξ*_ are the creation and annihilation operators for each plasmonic mode *ξ* with frequency *ω*_*ξ*_ where *n* plasmonic modes are considered. The losses due to each plasmonic mode are captured in eq. (1) through the creation and annihilation operates $a_{\xi }^{†}$ and *a*_*ξ*_, which act as jump operators with loss rate *κ*_*ξ*_ [22]. The raising and lowering operators for a QE with transition frequency *ω*_0_ are given by *σ* ^†^ and *σ*, respectively, with the position dependent coupling strength to each mode *ξ* expressed as *g*_*ξ*_(**r**), which depends on the QEs position within the plasmonic nanocavity.

**Figure 1:**
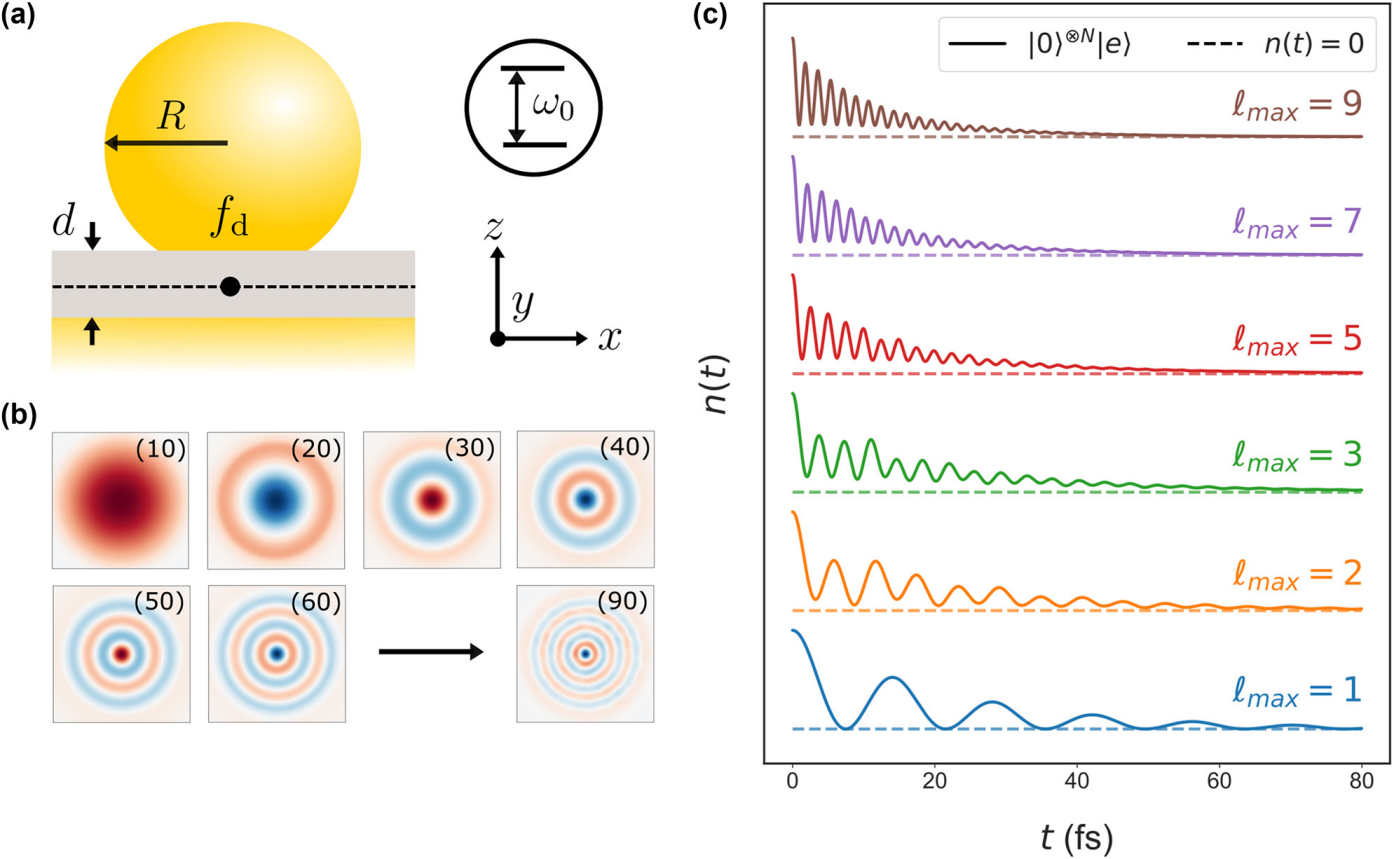
Multimode strong coupling. (a) Schematic of a gold nanoparticle on mirror (NPoM) cavity with radius *R* = 40 nm, facet diameter *f*_d_ = 16 nm, gap spacing *d* = 1 nm and gap permittivity *n*_gap_ = 2.5. A single QE is placed in the gap centre at **r**_0_ = (0, 0, 0). (b) Electric field, *E*_*z*_(*x*, *y*, 0), of the first nine *ξ* = (*ℓ*0) quasinormal modes (QNMs). (c) Evolution of the QEs excited state population, *n*(*t*) = |*c*_0_(*t*)|^2^, including interacting modes up to *N* = (*ℓ*_max_0) for various values of the index *ℓ*_max_.

One of the most common plasmonic systems that single molecule strong coupling has been realised in is the nanoparticle on mirror (NPoM) cavity [39], [40], [41] shown in Figure 1a. Here, we consider an NPoM cavity with nanoparticle radius *R* = 40 nm, circular facet size *f*_d_ = 16 nm, gap size *d* = 1 nm and gap refractive index *n*_gap_ = 2.5 – a single QE (two-level system) is placed within the gap at the nanocavity centre, i.e. at **r**_0_ = (0, 0, 0). Note that, although we focus on the NPoM cavity, the results in this paper are applicable to any system described by Eq. (1) and Eq. (2) regardless of the method of mode decomposition, provided the modes are non-interacting and have a Lorentzian lineshape.

The frequencies, loss rates and interaction strengths of the plasmonic modes – each interacting with the QE through Eq. (1) – are calculated classically using the auxiliary eigenvalue approach [42], [43], [44]. These are quasinormal modes (QNMs) with electric and magnetic fields denoted by ${\tilde{E}}_{\xi }\left(r\right)$ and ${\tilde{H}}_{\xi }\left(r\right)$ , respectively, and have a finite lifetime due to their complex eigenfrequency, which takes the form ${\tilde{\omega }}_{\xi }=\omega_{\xi }-i\frac{\kappa_{\xi }}{2}$ , where *ω*_*ξ*_ represents the resonant frequency and *κ*_*ξ*_ the mode decay rate. The coupling strength of each QNM *ξ* to the QE is given by: $g_{\xi }\left(r\right)=\sqrt{\frac{\omega_{\xi }}{\hbar {\text{V}}_{\xi }}}\mu \cdot {\tilde{E}}_{\xi }\left(r_{j}\right)$ where ***μ*** is the QE dipole moment and ${\text{V}}_{\xi }={∭}_{\Omega }\left[{\tilde{E}}_{\xi }\cdot \frac{\partial \omega \epsilon }{\partial \omega }{\tilde{E}}_{\xi }-\mu_{0}{\tilde{H}}_{\xi }\cdot \frac{\partial \omega \mu }{\partial \omega }{\tilde{H}}_{\xi }\right]dV$ is the QNM normalisation factor. In this system, QNMs are characterised according to their symmetries [30], [38] with each given an index *ξ* = (*ℓm*), where ℓ∈Z is the number of the modes radial anti-nodes and −*ℓ* ≤ *m* ≤ *ℓ* the pairs of azimuthal anti-nodes [30]. The electric field $E_{\xi }^{z}\left(x,y,0\right)$ of the *ξ* = (*ℓ*0) QNMs are shown in Figure 1b up to *ξ* = (90), while the corresponding frequencies, loss rates, normalised fields and coupling strengths of these modes are presented in the Supplementary information in Table 1. Importantly, the (*ℓ*0) modes are spherical and have their field maximum at the centre of the cavity, facilitating an environment for multimode strong coupling. In addition, the QE only interacts with the (*ℓ*0) modes since the electric field of all other modes vanishes at **r**_0_ = (0, 0, 0).

To determine the effect of multiple modes on the quantum dynamics, we first consider a QE in the initial state *c*_0_(0) = |0⟩^⊗*n*^|*e*⟩ with dipole moment $\mu =72\;\hat{z}\;\text{D}$ [45] and transition frequency resonant with the (10) mode, i.e. *ω*_0_ = *ω*_(10)_ = 283 THz. The quantum dynamics when including interacting modes up to *n* = (*ℓ*_max_0) are shown in Figure 1c up to *ℓ*_max_ = 9. Note that intermediate steps with *l*_max_ < 9 illustrate the effect of gradually including more modes coupled to the QE. For a single mode (*ℓ*_max_ = 1), the QE exchanges energy with the cavity at a single (Rabi) frequency, which experiences large damping rate due to the high plasmonic losses as expected. However, when two modes interact with the QE (*ℓ*_max_ = 2) – with one mode off resonant – the quantum dynamics become increasingly more complex. In this case, the excited state population exhibits multiple oscillations arising from the different coupling strengths and detunings of each mode. In addition, the dominant oscillations in the signal are faster than with a single mode alone. The quantum dynamics continue to exhibit additional multiple oscillation frequencies until, for this system, approximately five modes (*ℓ*_max_ ∼ 5) are considered. From this point onwards, a single ultra-fast oscillation frequency dominates the QEs evolution. The energy exchange with the multimode plasmonic nanocavity is almost one order of magnitude faster than when using a single mode approximation. Therefore, from Figure 1c, it is obvious that off-resonant modes have a significant impact on the quantum dynamics and must always be taken into account to accurately describe strong coupling in such systems. In addition, the populations of each mode are shown in the *Mode amplitudes* section in the Supplementary information, where the off-resonant modes are determined to have the largest amplitude at this dipole moment.

### 2.1 Quantum oscillations in multimode strong coupling

The oscillations shown in Figure 1c arise from the off-resonant modes but are hard to interpret due to the high plasmonic losses. To overcome this, we initially assume the system evolves in the absence of loss (i.e. *κ*_*ξ*_ = 0) such that the evolution is unitary and governed by the Schrödinger Equation $i\partial_{t}|\psi 〉=H|\psi 〉$ where the excited state population of the QE can be determined both numerically and analytically. This helps provide a comprehensive and in-depth understanding of the interactions involved. To derive an equation for the QEs excited state population, we first transform to the interaction picture such that $i\partial_{t}|\tilde{\psi }〉=V_{\text{int}}|\tilde{\psi }〉$ where $V_{\text{int}}={\text{e}}^{\text{i}H_{0}t}H_{\text{int}}{\text{e}}^{-\text{i}H_{0}t}$ and $|\tilde{\psi }〉={\text{e}}^{\text{i}H_{0}t}|\psi 〉$ . In fact, due to conservation of the excitation number, we can also express the quantum state as $|\tilde{\psi }〉=c_{0}|0,e〉+\sum_{j}c_{\xi }a_{\xi }^{†}|0,g〉$ with amplitudes *c*_0_ and *c*_*ξ*_, respectively. From this, we obtain two coupled equations of motion:

(3) $$i\partial_{t}c_{0}=\underset{\xi }{\sum }g_{\xi }c_{\xi }{\text{e}}^{\text{i}\Delta_{\xi }t}$$

(4) $$i\partial_{t}c_{\xi }=g_{\xi }c_{0}{\text{e}}^{-\text{i}\Delta_{\xi }t}$$

where Δ_*ξ*_ = *ω*_0_ − *ω*_*ξ*_ is the detuning between the QE and mode *ξ*. To solve Eq. (3) and Eq. (4) for *c*_0_(*t*), we take their Laplace Transform, where we define $\tilde{c}\left(s\right)=L\left[c\left(t\right)\right]$ and use the identities $L\left[\partial_{t}x\right]=s\tilde{x}\left(s\right)-x_{0}\left(0\right)$ and $L\left[f\left(t\right){\text{e}}^{\alpha t}\right]=\tilde{f}\left(s-\alpha \right)$ . Hence, two new coupled equations are obtained given by $s{\tilde{c}}_{0}\left(s\right)-c_{0}\left(0\right)=-i\sum_{\xi }g_{\xi }{\tilde{c}}_{\xi }\left(s-i\Delta_{\xi }\right)$ and $s{\tilde{c}}_{\xi }\left(s\right)-c_{\xi }\left(0\right)=-ig_{\xi }{\tilde{c}}_{0}\left(s+i\Delta_{\xi }\right)$ , respectively, which we then solve algebraically to give:

(5) $${\tilde{c}}_{0}\left(s\right)={\left[s+\overset{n}{\underset{\xi }{\sum }}\frac{g_{\xi }^{2}}{\left(s-i\Delta_{\xi }\right)}\right]}^{-1}c_{0}\left(0\right)$$

where *c*_0_(0) = 1 is the initial population of the QEs excited state. The key step in solving Eq. (5) is to write the expression in square brackets as a quotient of two polynomial functions:

(6) $${\left[s+\overset{n}{\underset{j}{\sum }}\frac{g_{j}^{2}}{\left(s-i\Delta_{j}\right)}\right]}^{-1}=\frac{{\text{Q}}^{\left(n\right)}\left(s\right)}{{\text{P}}^{\left(n+1\right)}\left(s\right)}$$

where

(7) $${\text{P}}^{\left(n+1\right)}\left(s\right)=s\overset{n}{\underset{j}{\prod }}\left(s-i\Delta_{j}\right)+\underset{j}{\sum }g_{j}^{2}\underset{k\neq j}{\prod }\left(s-i\Delta_{k}\right)$$

(8) $${\text{Q}}^{\left(n\right)}\left(s\right)=\overset{n}{\underset{j}{\prod }}\left(s-i\Delta_{j}\right)$$

Importantly, when Δ_*i*_ ≠ Δ_*j*_ for all *i* ≠ *j*, then P^(*n*+1)^(*s*) has *n* + 1 distinct and purely imaginary roots *iλ*_*j*_ where *λ*_*n*_ < *λ*_*n*−1_ < … < *λ*_1_ < *λ*_*n*+1_ (see *Proof of distinct roots section* in Supplementary information for more details). This enables us to factorise P^(*n*+1)^(*s*) and rewrite Eq. (5) in a form that has a simple inverse Laplace Transform:

(9) $$\frac{{\text{Q}}^{\left(n\right)}\left(s\right)}{{\text{P}}^{\left(n+1\right)}\left(s\right)}=\overset{n+1}{\underset{j=1}{\sum }}\frac{\alpha_{j}}{s-i\lambda_{j}}$$

where $\alpha_{j}=\text{Q}\left(i\lambda_{j}\right)/\frac{d{\text{P}}^{\left(n+1\right)}}{ds}{|}_{i\lambda_{j}}$ . Finally, taking the inverse Laplace Transform of Eq. (9) now yields an expression for *c*_0_(*t*), from which we find the QEs excited state population:

(10) $$\begin{array}{ll} |c_{0}\left(t\right){|}^{2} & =\overset{n+1}{\underset{j=1}{\sum }}\alpha_{j}^{2}+2\alpha_{n+1}\overset{n}{\underset{j=1}{\sum }}\alpha_{j}\cos\left(\Omega_{j}t\right)\\ & \;+\overset{n}{\underset{j=1}{\sum }}\underset{k\neq j}{\sum }\alpha_{j}\alpha_{k}\cos\left(\left(\Omega_{j}-\Omega_{k}\right)t\right) \end{array}$$

where Ω_*j*_ = *λ*_*j*_ − *λ*_*n*+1_ and, therefore, gives the oscillation frequencies present in the quantum dynamics of a multimode system in the limit of zero loss. In fact, Eq. (10) shows that there are up to *n*(*n* + 1)/2 frequency components in the excited state population of a QE strongly coupled with *n* modes. More specifically, there are *n* components corresponding to the mode frequencies |Ω_*j*_| and *n*(*n* − 1)/2 corresponding to the interference terms |Ω_*j*_ − Ω_*i*_|. Importantly, the oscillation frequencies |Ω_*j*_ − Ω_*i*_| arise from non-interacting modes (of a Lorentzian form) and, therefore, are not equivalent to the direct coupling between modes as seen in hybrid metallodielectric cavities (that may exhibit modes of non-Lorentzian form).

**Figure 2:**
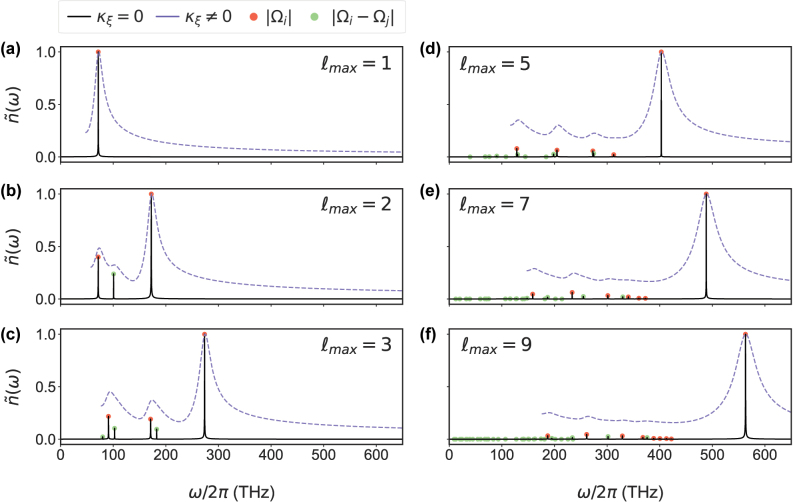
Frequency components in multimode strong coupling. The Fourier Transform $\tilde{n}\left(\omega \right)$ of the QEs excited state population when including interacting modes up to *ξ* = (*ℓ*_max_0). The solid black lines are numerical results without loss (i.e. *κ*_*ξ*_ = 0) and the dashed lines with plasmonic loss (i.e. *κ*_*ξ*_ ≠ 0). The red and green dots result from |Ω_*j*_| and |Ω_*i*_ − Ω_*j*_| calculated from the roots of Eq. (7). The sub-figures show the frequencies for when (a) *ℓ*_max_ = 1, (b) *ℓ*_max_ = 2, (c) *ℓ*_max_ = 3, (d) *ℓ*_max_ = 5, (e) *ℓ*_max_ = 7, (f) *ℓ*_max_ = 9, respectively. Note, the spectra with loss are cut to remove the peak at *ω* = 0, which results from the FT of a damped oscillations.

The oscillation frequencies |Ω_*j*_| and |Ω_*j*_ − Ω_*i*_| – which are calculated through the roots of Eq. (7) – are shown in Figure 2 in red and green dots, respectively. They are found to show excellent agreement with the full numerical calculations, which show the Fourier Transform (FT) of the quantum dynamics calculated with no loss (*κ*_*ξ*_ = 0) through unitary evolution (black lines) and with loss (*κ*_*ξ*_ ≠ 0 obtained from the QNM calculations for each mode) through integration of Eq. (1) (purple dashed lines). Intermediate steps with *l*_max_ < 9 illustrate the effect of gradually including more modes coupled to the QE. Such a systematic study elucidates the origin of each oscillation frequency and, therefore, helps in understanding the physics of multimode cavities governed by Eq. (1) or Eq. (10). In addition, note that the broadening of the peaks due to loss also has the effect that if the oscillation frequencies are spectrally close, it becomes difficult to resolve them in the spectra as seen in Figure 2.

We initially consider one plasmonic mode (*ℓ*_max_ = 1) as is often assumed to be the case and obtain the Rabi oscillation frequency |Ω_1_| as expected. Considering two plasmonic modes (*ℓ*_max_ = 2) leads to three distinct peaks, one corresponding to each mode, i.e. |Ω_1_| and |Ω_2_| and one interference peak |Ω_1_ − Ω_2_| of smaller amplitude. Here, all three peaks have a high intensity and contribute to the QEs evolution, but the off-resonant mode is the most significant. This is not always the case, and as we see later depends on the number and properties of the strongly coupled modes (i.e. their coupling strengths and detunings). As we increase the number of modes strongly coupled with the QE, we continue to observe up to *n*(*n* + 1)/2 oscillations in the quantum dynamics. For a large number of modes (i.e. *ℓ*_max_ ≥ 5), the quantum dynamics are completely dominated by a single ultra-fast frequency component |Ω_*n*_| with only a small contribution from other components. It is extremely important to note, that although we associate the frequency component |Ω_*j*_| with the mode *j*, each peak still depends on all the other strongly coupled modes in the cavity. In fact, the |Ω_*j*_|, mode *j* association is lost the more strongly coupled modes are considered, as each mode gradually shifts from being a distinct peak to contributing to the single ‘supermode’ frequency component |Ω_*n*_|.

Note that this ultra-fast oscillation |Ω_*n*_| emerges from the combined effect of all modes and does not occur with the same amplitude and frequency if only some of the modes are considered (see Supplementary information Figure S2). Therefore, this is a collective phenomenon and necessitates a full description of the plasmonic environment. We also note that the supermode behaviour described here arises from the collective and coherent response to multiple (potentially distinct) modes and is different from the apparent single mode behaviour arising from damping or dissipation, which prevents multiple modes that are spectrally close from being resolved. In addition, this ultrafast oscillation frequency – due to the collective multimode strong coupling – is also robust to displacements of the QE as shown in the Supplementary information Figure S7 and S8. This is due to the large field enhancements of the *m* ≠ 0 modes away from the centre.

### 2.2 Critical transition to collective strong coupling

**Figure 3:**
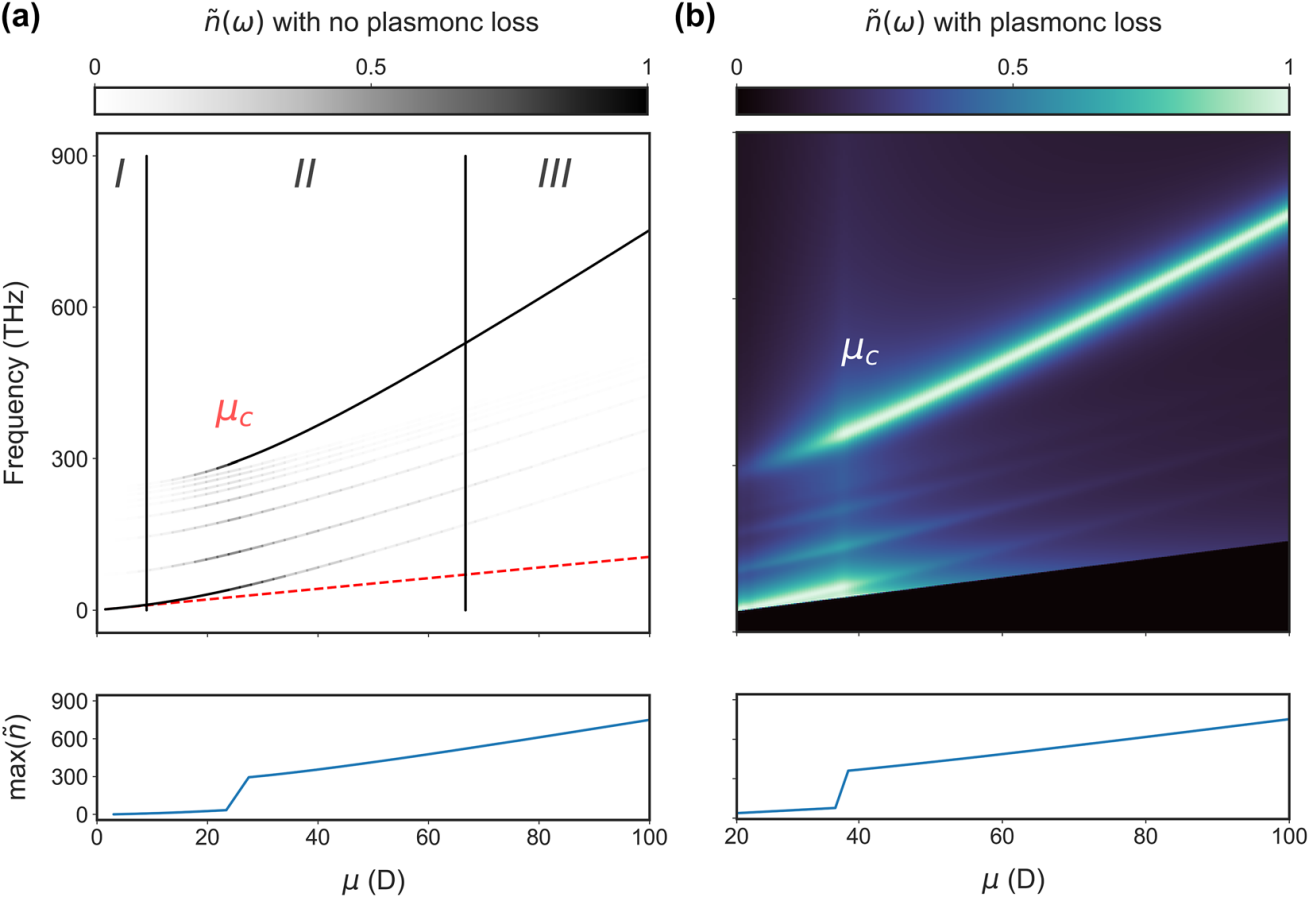
Transition from single to collective multimode strong coupling. Fourier Transform of the QEs excited state population as a function of dipole moment *μ*. The QE interacts with modes up to *ξ* = (90) with (a) assuming no loss, i.e. *κ*_*ξ*_ = 0 and (b) including loss, i.e. *κ*_*ξ*_ ≠ 0. Three regions, dictated by the dipole moment, govern the QEs evolution (*I*) single mode strong coupling (*II*) multimode strong coupling and (*III*) collective multimode strong coupling. The single mode approximation is shown in the dashed red dashed line. The critical dipole moment is also shown and represents the onset of the ultra-fast collective strong coupling. The FT spectra in (b) are cut to remove the peak at *ω* = 0, which results from the FT of damped oscillations. Below 20 D, the |Ω_1_| peak significantly overlaps with the *ω* = 0 peak and so cannot be removed successfully.

The amplitudes of the oscillations at frequencies |Ω_*j*_| and |Ω_*j*_ − Ω_*i*_| depend on the magnitude of the coupling strengths *g*_*j*_ compared to the detunings Δ_*j*_. To determine the impact of the coupling strength on the oscillations reported above, we change the dipole moment *μ* (which is an overall scale factor of the coupling strengths) and identify three distinct regions. In Figure 3a and b, the FT of the quantum dynamics is shown for a system with and without loss, respectively, for many dipole moments. For small dipole moments such that *g*_*i*_ < |Δ_*i*_ − Δ_*i*+1_| for each mode (region *I*), the dynamics are determined by a single resonant mode and leads to a single (Rabi) oscillation. This applies to the majority of molecules commonly utilised in plasmonics, such as Cy5 and methylene blue, indicating that for QEs with small *μ* single mode strong coupling is reached. Note, however, that when plasmonic losses are present in this system, damping prevents single mode strong coupling even for the same dipole moments. For intermediate dipole moments (i.e. quantum dots) such that |Δ_*i*_ − Δ_*i*+1_|^2^ < |*g*_*i*_|^2^ ≪|Δ_*i*+1_||Δ_*i*_ − Δ_*i*+1_| (region *II*), then multimode strong coupling occurs. In this region, the off-resonant modes produce multiple distinct frequency peaks |Ω_*j*_|, while the interference components |Ω_*i*_ − Ω_*j*_| have a small amplitude and are hard to observe (further discussion is included in the Supplementary information Figure S3 for *μ* = 30 D). Finally, for large dipole moments such that |*g*_*i*_|^2^ ≫|Δ_*i*+1_||Δ_*i*_ − Δ_*i*+1_| (region *III*), collective multimode strong coupling occurs. While this is a multimode phenomenon, the quantum dynamics are now governed by a single ultra-fast ‘supermode’ oscillation |Ω_*n*_|. This ‘supermode’ oscillation results in energy exchange over an order of magnitude faster than single mode strong coupling alone. In region *II*, the supermode peak |Ω_*n*_| can have the largest amplitude at some dipole moments, but in region *III*, it completely dominates the quantum dynamics. The quantum dynamics in Figures 1 and 2 for *μ* = 72 D are within region *III* and, therefore, show the collective interaction with multiple off-resonant modes.

Crucially, a transition occurs at a critical dipole moment (*μ*_c_), where the dominant frequency component switches from |Ω_1_| to |Ω_*n*_| as shown in Figure 3 (bottom panels). At the critical dipole moment, the maximum oscillation frequency in a lossless system increases from 34 to 294 THz at *μ*_*c*_ ∼ 27 D and from 75 to 360 THz at *μ*_*c*_ ∼ 38 D in an equivalent system with loss. The critical dipole moment occurs at higher values with loss because dissipation suppresses the amplitude of the off-resonant peaks and so requires larger coupling to reach criticality. In the limit of large coupling, the oscillation amplitudes *α*_*i*_ switch from behaving like ${\left[1+\lambda_{i}\sum_{j=1}^{n}1/\left(\lambda_{i}-\Delta_{j}\right)\right]}^{-1}$ to ${\left[1+\lambda_{i}\sum_{j=1}^{n}1/\left(\lambda_{i}-\Delta_{j}\right)-\sum_{j\neq k}g_{k}^{2}/\left(\left(\lambda_{i}-\Delta_{j}\right)\left(\lambda_{i}-\Delta_{k}\right)\right)\right]}^{-1}$ (see *Dependence of amplitudes on dipole moment* section in the Supplementary information for more details). The former has the most dominant oscillations for *λ*_1_, i.e. the component oscillating at |Ω_1_|, while the latter has the most dominant oscillations for *λ*_*n*_, i.e. the component oscillating at |Ω_*n*_|. This occurs because the interactions with each mode interfere constructively for the amplitude *α*_*n*_ when *μ* > *μ*_*c*_, whilst there is always some destructive interference for the other amplitudes. Therefore, past the critical dipole moment (which occurs at some point between these two regions) energy exchange with the plasmonic nanocavity becomes ultra-fast, and represents a collective multimode strong coupling regime. This can also be observed in the mode populations, where: (1) below the critical dipole moment most modes oscillate out of phase with one another and (2) above the critical dipole moment they begin to oscillate in phase at the supermode frequency |Ω_*n*_|. For more information and discussion, see *Mode amplitudes* section in the Supplementary information.

This transition can be observed experimentally by measuring the scattering cross section spectra of the system – which has resonances at the eigenenergies of the Hamiltonian. In a bare cavity (without the QE), these experimental spectral resonances occur at the mode frequencies *ω*_*ξ*_. However, with the presence of a QE strongly coupled with just the resonant plasmonic mode (in region *I*), the (10) mode peak splits into two polariton branches with splitting |Ω_1_| characteristic of single-mode strong coupling as previously reported experimentally [5]. For collective multimode strong coupling (region *III*), the largest splitting occurs between the lowest and highest eigenfrequencies, with splitting |Ω_*n*_|. In the Supplementary information Figure S10, we present the eigenenergies of the Hamiltonian as a function of dipole moment, as a useful guide to experiments on the expected mode splitting, but one can obtain theoretically the full scattering cross-sectional spectrum via quantum simulations similar to those presented in [39] for a single mode. Nevertheless, we expect experimental spectra to have consistent linewidths, amplitudes and resonant frequencies as the results presented in Figures 2 and 3b.

In plasmonic systems, multiple plasmonic modes cannot be neglected; the single-mode approximation holds only for small dipole moments, where strong coupling is often unachievable in practical setups due to the large plasmonic losses. As the coupling strength increases, the interaction transitions into a multimode interaction and eventually to a fully collective regime characterised by a dominant ultra-fast oscillation governing the quantum dynamics. This behaviour highlights a key advantage of quantum dots (QDs) to reach the collective multimode strong coupling regime in quantum plasmonics; having dipole moments varying significantly and even surpassing 70D (as seen in In GaN/GaN QDs). As a result, QDs can produce ultra-fast oscillations nearly two orders of magnitude faster than those achievable with dye molecules, and one order of magnitude faster than single mode approximations. In addition, collective multimode strong coupling can also be attained in plasmonic systems of different shapes and with further reduced mode volumes, enabling critical coupling even at lower dipole moments. It should be noted that our results are general for any nanophotonic system that supports multiple Lorentzian modes, since their spectral proximity allows for multiple modes to be coupled to a QE. These findings present a novel pathway for ultrafast coupling in multimode systems, with significant potential for applications in quantum information and sensing.

## 3 Conclusions

In conclusion, we have demonstrated the crucial importance of off-resonant plasmonic modes strongly coupled to a QE. We show that *n* strongly coupled plasmonic modes introduce up to *n*(*n* + 1)/2 oscillation frequencies in the excited state population of a single QE going far beyond the single mode approximation. Additionally, we identify three distinct coupling regimes based on increasing dipole moment: single-mode strong coupling, multimode strong coupling and collective multimode strong coupling. These oscillations are influenced by the number of modes, their coupling strengths and detunings. The results deepen our understanding of quantum dynamics in multimode environments and demonstrate the potential for plasmonic nanocavities to achieve ultra-fast energy transfer in light-driven quantum technologies.

## Supplementary Material

Supplementary Material Details
