# Supplementary material for: Collective multimode strong coupling in plasmonic nanocavities
Source: Nanophotonics. 2025 Mar 21;14(11):2065–73. doi: 10.1515/nanoph-2024-0618 (PMC12133250; doi:10.1515/nanoph-2024-0618)
Supplement: Supplementary file 1 — Supplementary Material Details [file j_nanoph-2024-0618_suppl_001.pdf]

Collective multimode strong coupling in plasmonic nanocavities - Supplementary Information

Angus Crookes[1], Ben Yuen[1], and Angela Demetriadou*[1]

[1] *School of Physics and Astronomy, University of Birmingham, Edgbaston, Birmingham, B15 2TT, United Kingdom*

February 18, 2025

Quasinormal Mode Formulation of Plasmonic Resonators

The quasinormal modes (QNMs) for a plasmonic system can be found by solving the source-free, time-harmonic Maxwell equations - which take the form of a general non-linear eigenvalue problem:

$$\begin{bmatrix} 0 & i\epsilon^{-1}(\mathbf{r},\tilde{\omega})\nabla\times \\ -i\mu^{-1}(\mathbf{r},\tilde{\omega})\nabla\times & 0 \end{bmatrix} \begin{bmatrix} \tilde{\mathbf{E}}(\mathbf{r}) \\ \tilde{\mathbf{H}}(\mathbf{r}) \end{bmatrix} = \tilde{\omega} \begin{bmatrix} \tilde{\mathbf{E}}(\mathbf{r}) \\ \tilde{\mathbf{H}}(\mathbf{r}) \end{bmatrix} \tag{S1}$$

where $\tilde{\omega}$ is the QNM frequency, ϵ and μ the dispersive permittivity and permeability, and $\tilde{\mathbf{E}}$ and $\tilde{\mathbf{H}}$ satisfy an outgoing wave boundary condition. Here, we use a two-pole Drude-Lorentz function to model the permittivity of gold:

*a.demetriadou@bham.ac.uk

$$\epsilon_{\mathrm{Au}}(\omega) = \epsilon_\infty \left(1 + \sum_{j=1}^{2} \frac{\omega_{\mathrm{p},j}^2}{\omega_{0,j}^2 - \omega^2 - i\gamma_j\omega}\right) \tag{S2}$$

where $\omega_{\mathrm{p},1} = 5.37 \times 10^{15}$ rad/s, $\omega_{\mathrm{p},2} = 2.636 \times 10^{15}$ rad/s, $\omega_{0,1} = 0$ rad/s, $\omega_{0,2} = 4.572 \times 10^{15}$, $\gamma_1 = 6.22 \times 10^{13}$ rad/s and $\gamma_2 = 1.332 \times 10^{15}$ are the plasma frequency, resonant frequency and damping rate of each pole obtained from [1] and $\epsilon_\infty = 6\epsilon_0$ is the asymptotic permittivity. When the permittivity is described by Eq. (S2) the non-linear eigenvalue problem in Eq. (S1) can be made linear by introducing a pair of auxiliary fields (one for each pole) as:

$$\tilde{\mathbf{P}}_{i,j}(\mathbf{r}) = \frac{\epsilon_\infty \omega_{\mathrm{p},j}^2}{\omega_{0,j}^2 - \tilde{\omega}_i^2 - i\gamma_j\tilde{\omega}_i}\tilde{\mathbf{E}}(\mathbf{r}), \qquad \tilde{\mathbf{J}}_{i,j}(\mathbf{r}) = -i\tilde{\omega}_i\tilde{\mathbf{P}}_{i,j}(\mathbf{r}) \tag{S3}$$

where $\tilde{\mathbf{P}}_{i,j}(\mathbf{r})$ and $\tilde{\mathbf{J}}_{i,j}(\mathbf{r})$ are the auxiliary polarisation and current fields of the ith QNM and j^{th} pole. This results in a linear eigenvalue equation given by:

$$\begin{bmatrix} 0 & -i\mu_0^{-1}\nabla\times & 0 & 0 & 0 & 0 \\ i\epsilon_0^{-1}\nabla\times & 0 & 0 & -i\epsilon_\infty^{-1} & 0 & -i\epsilon_\infty^{-1} \\ 0 & 0 & 0 & i & 0 & 0 \\ 0 & i\omega_{\mathrm{p},1}^2\epsilon_\infty & -i\omega_{0,1}^2\epsilon_\infty & -i\gamma_1 & 0 & 0 \\ 0 & 0 & 0 & 0 & 0 & i \\ 0 & i\omega_{\mathrm{p},2}^2\epsilon_\infty & 0 & 0 & -i\omega_{0,2}^2\epsilon_\infty & -i\gamma_2 \end{bmatrix} \begin{bmatrix} \tilde{\mathbf{H}}_i \\ \tilde{\mathbf{E}}_i \\ \tilde{\mathbf{P}}_{i,1} \\ \tilde{\mathbf{J}}_{i,1} \\ \tilde{\mathbf{P}}_{i,2} \\ \tilde{\mathbf{J}}_{i,2} \end{bmatrix} = \tilde{\omega}_i \begin{bmatrix} \tilde{\mathbf{H}}_i \\ \tilde{\mathbf{E}}_i \\ \tilde{\mathbf{P}}_{i,1} \\ \tilde{\mathbf{J}}_{i,1} \\ \tilde{\mathbf{P}}_{i,2} \\ \tilde{\mathbf{J}}_{i,2} \end{bmatrix} \tag{S4}$$

where a full description can be found in [2]. The full QNM analysis is found by implementing *QNMEig* to solve equation (S4)- an open-source, efficient finite element solver for the rigorous analysis of complicated plasmonic resonators. From

this, we obtain complex eigenfrequencies and eigenvectors (near-field distributions) of the QNMs supported by the linearised system shown in Eq. (S4).

Nanoparticle on Mirror Quasinormal modes

Figure S1 (a) shows the QNM field distributions $E_z^{\xi}(x, y, 0)$ within the gap of the nanoparticle on mirror cavity. It is clear that only the $m = 0$ modes have a non-zero field at the centre, and so are the only modes considered in the main text. Further details on the nanoparticle on mirror QNMs can be found in [3].

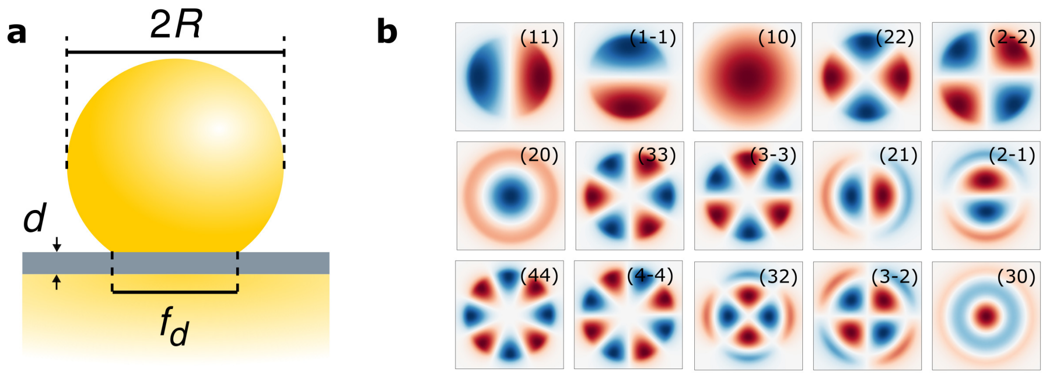

Figure S1: (a) Schematic of a gold nanoparticle on mirror (NPoM) cavity with radius $R = 40$ nm, facet diameter $f_{\rm d} = 16$ nm, gap spacing $d_{\rm gap} = 1$ nm, and gap permittivity $n_{\rm gap} = 2.5$. (b) The electric field $E_{\xi}^{z}(x, y, z)$ of the first fifteen QNMs supported by the nanoparticle on mirror.

The frequencies, loss rates, and coupling strengths of each cylindrical $\xi = (\ell 0)$ mode are shown in Table 1. In particular, the coupling strengths are calculated at two dipole moments $\mu_0 = 72$ D and the critical dipole moment $\mu_{\rm c} =$ D.

ξ	(10)	(20)	(30)	(40)	(50)	(60)	(70)	(80)	(90)
ω_ξ (eV)	1.179	1.468	1.746	1.913	2.009	2.057	2.107	2.148	2.178
Δ_ξ (eV)	0	0.29	0.567	0.734	0.83	0.878	0.928	0.969	0.999
κ_ξ (eV)	0.061	0.065	0.082	0.110	0.147	0.164	0.160	0.169	0.180
$\tilde{\mathbf{E}}_\xi(\mathbf{r}_0)/\sqrt{\mathrm{QN}_\xi}$	2.28	3.86	4.67	4.91	4.43	4.09	4.72	4.63	4.30
$g(\mathbf{r}_0,\ \mu_0)$ (eV)	0.148	0.281	0.371	0.408	0.377	0.352	0.411	0.407	0.381
$g(\mathbf{r}_0,\ \mu_\mathrm{c})$ (eV)	0.078	0.148	0.196	0.216	0.199	0.186	0.217	0.215	0.201

Table 1: Table giving the frequencies, loss rates, detunings, coupling strengths, and normalised fields (in units of $10^{17}N/JC$) of each $\xi = (\ell 0)$ plasmonic mode. The coupling strengths are calculated at the QE position $\mathbf{r}_0 = (0,0,0)$ and for dipole moments $\mu_0 = 72$ D and the critical dipole moment μ_c where $g(\mathbf{r}_0, \mu)$ is the magnitude of the coupling strength at position $\mathbf{r}$ and dipole moment μ.

Proof of distinct roots

In the derivation for the excited state population of a QE interacting with n modes, we define the polynomial function:

$$\mathrm{P}^{(n+1)}(s) = s\prod_j^n (s - i\Delta_j) + \sum_j g_j^2 \prod_{k\neq j}(s - i\Delta_k) \tag{S5}$$

which we claim has $n+1$ distinct roots. To prove this statement, we define the complex representation of the polynomial in eq. (S5) as:

$$\mathcal{P}^{(n+1)} = -i^{n+1}\mathrm{P}^{(n+1)}(is) \tag{S6}$$

$$= s\prod_j^n (s - \Delta_j) - \sum_j g_j^2 \prod_{k\neq j}(s - \Delta_k) \tag{S7}$$

where we multiply by the factor i^{n+1} to remove additional constant factors of i. We then determine the roots of eq. (S7) by evaluating the polynomial at the different

mode detunings i.e. $\mathcal{P}^{(n+1)}(\Delta_j)$. This gives the expression:

$$\mathcal{P}^{(n+1)}(\Delta_j) = -g_j^2 \prod_{k=1}^{j-1} (\Delta_j - \Delta_k) \prod_{k=j+1}^{n} (\Delta_j - \Delta_k) \tag{S8}$$

$$= (-1)^j g_j^2 \prod_{k \neq j}^{n} |\Delta_j - \Delta_k| \tag{S9}$$

where we have first separated the polynomial into a product of two expressions: (left) those with $\omega_j > \omega_k$ and (right) those with $\omega_j < \omega_k$. Therefore, since $\Delta_n < \Delta_{n-1} ... \Delta_2 < \Delta_1 \leq 0$, the second product is always positive, while we gain a factor of (-1) for each term in the left product. This results in the expression in eq. (S9) when we explicitly factor out the sign changes. Note, in deriving eq. (S9) we have assumed that $\Delta_j \neq \Delta_k$. Importantly, we can now see that the sign of eq. (S9) depends on the mode detuning at which we evaluate the polynomial. Using this information, along with the behaviour of the function at $\pm\infty$ i.e. $\text{sgn}\left(\mathcal{P}^{(n+1)}(-\infty)\right) = (-1)^{n+1}$ and $\text{sgn}\left(\mathcal{P}^{(n+1)}(+\infty)\right) = 1$, we find that the sign of $\mathcal{P}^{(n+1)}(s)$ changes as shown in Table 2.

s	$-\infty$	Δ_n	Δ_{n-1}	...	Δ_1	$+\infty$
odd n	$+$	$-$	$+$	...	$-$	$+$
even n	$-$	$+$	$-$	...	$-$	$+$

Table 2: Table demonstrating that $\mathcal{P}^{(n+1)}(s)$ has $n+1$ sign changes, and therefore $n+1$ real and distinct roots. The number of roots is the same for odd and even numbers of modes.

Importantly, for any number of modes n, there are always $n+1$ sign changes in the polynomial $\mathcal{P}^{(n+1)}(s)$. Therefore, $\mathcal{P}^{(n+1)}(s)$ has $n+1$ real distinct roots given by λ_j that lie between the detunings of each mode. Note we define these roots such that $\lambda_n < \lambda_{n-1} ... < \lambda_1 < \lambda_{n+1}$ where all $\lambda_j < 0$ for $j \neq n+1$ and $\lambda_{n+1} > 0$. Finally, since $\mathcal{P}^{(n+1)}(s)$ is the complex representation of $\mathrm{P}^{(n+1)}(s)$ we can write the

$n+1$ roots of $\mathrm{P}^{(n+1)}(s)$ as $i\lambda_j$. This allows us to factorise the polynomial and find the inverse Laplace Transform.

Collective multimode strong coupling

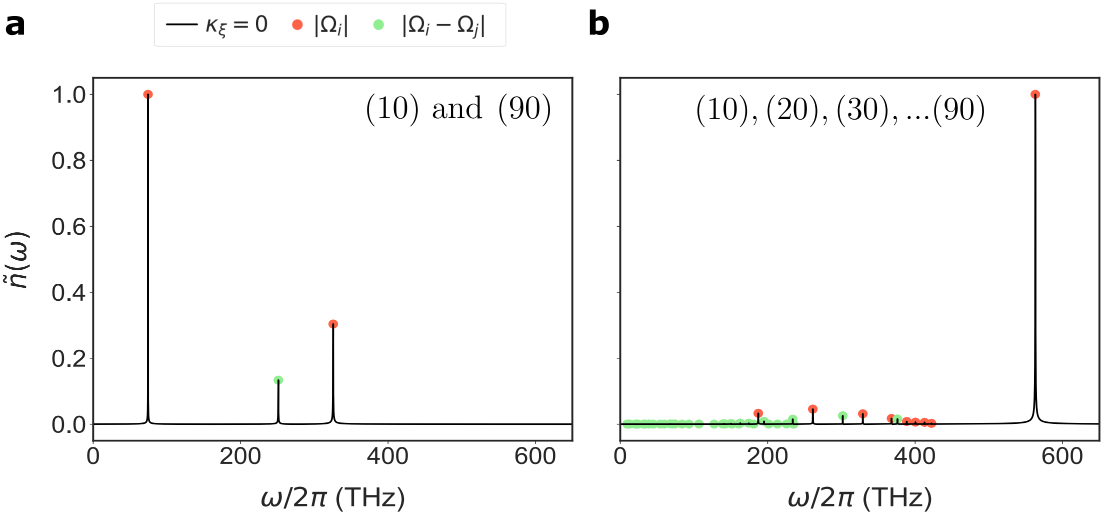

Figure S2: Frequency components in the QEs excited state population when interacting with the modes (a) (10) and (90) and (b) (10) $\rightarrow$ (90). The collective effect of multiple strongly coupled modes is an ultra-fast oscillation $|\Omega_n|$.

In Figure S2, we present frequency components in the excited state population of a quantum emitter at $\mathbf{r}_0 = (0,0,0)$ interacting with (a) just the (10) and (90) modes and (b) the $(10),(20),(30),...(90)$ modes. The QE is resonant with the (10) mode i.e. $\omega_0 = \omega_{(10)}$ and has dipole moment $\mu = 72$ D as in the main text. Figure S2 shows that the ultra-fast oscillations would not occur with the same amplitude and frequency if the only modes were (10) and (90). Therefore, frequency components are highly collective, and requires a full consideration of the plasmonic environment. The collective nature of the frequency components is also

shown in the next section, where we consider a quantum emitter with a smaller dipole moment.

Quantum Dynamics and Fourier Transform

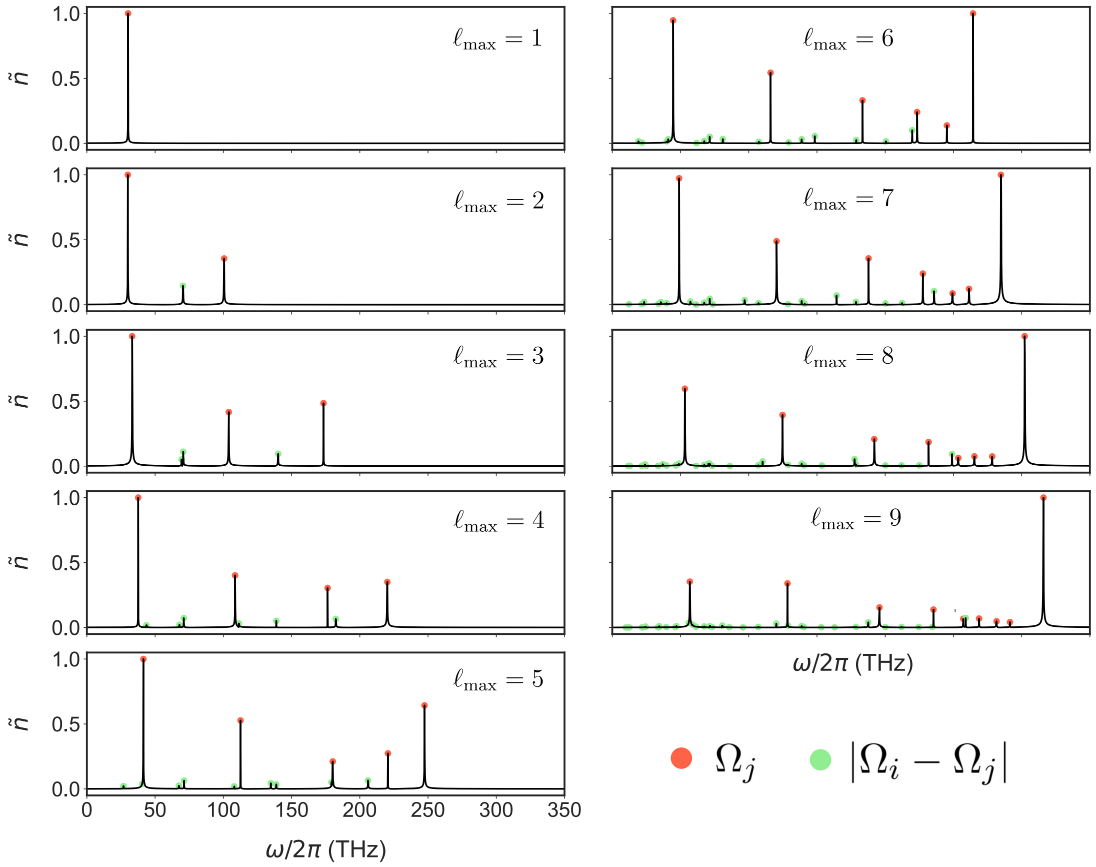

Figure S3: Frequency components in multimode strong coupling for a QE with dipole moment $\mu =$. The Fourier Transform of the QEs excited state population when including interacting modes up to $\xi = (\ell_{\max}0)$ are shown. The solid black lines are numerical results without loss (i.e. $\kappa_\xi = 0$) and the red and green dots are $|\Omega_j|$ and $|\Omega_i - \Omega_j|$ calculated from the roots of $\mathrm{P}^{(n+1)}(s)$.

Here, we present the FT of the quantum dynamics of a single quantum emitter at $\mathbf{r}_0 = (0, 0, 0)$ interacting with the $\xi = (\ell 0)$ modes of the plasmonic nanocavity. Note that here, the quantum emitter has a smaller dipole moment than in the main text i.e. $\mu = 30$ D which changes the impact of off-resonant modes on the frequencies. This is shown explicitly in the Fourier Transform in Figure S3. Note, the frequencies shown here are without loss i.e. $\kappa_\xi = 0$.

Comparison between numerical and analytical results

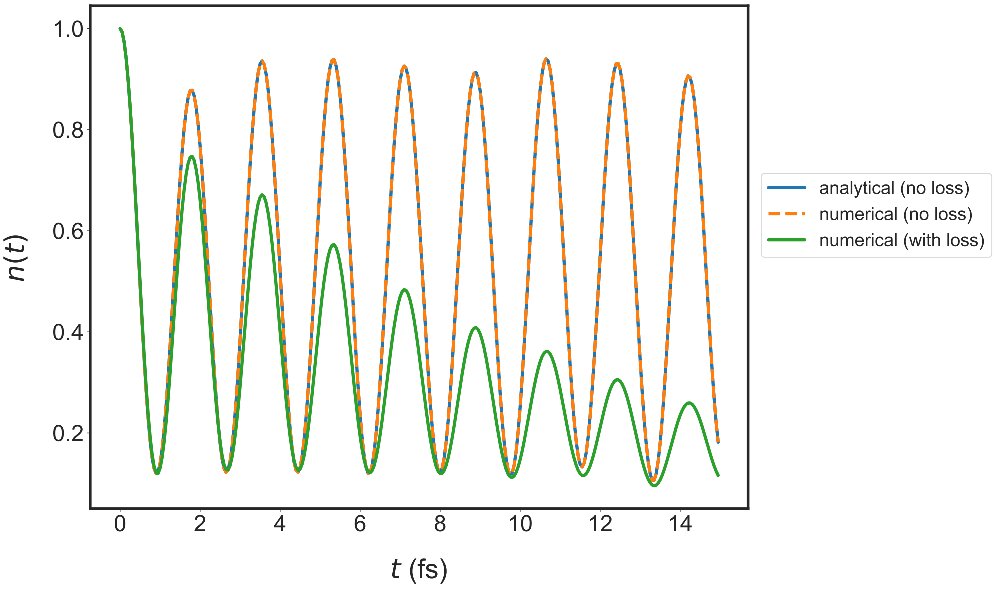

Figure S4: Comparison between the quantum dynamics calculated using both analytical and numerical methods.

Here, we show a comparison between the quantum dynamics calculated using: (1) Eq. (10) in the main text i.e. analytically and with no loss, (2) numerically with no loss, and (3) numerically with loss. The analytical and numerical methods without loss show exact agreement. The oscillation frequencies using (3) agree with (2) and (1) .

Mode amplitudes

The relative amplitudes of the different modes $\xi = (\ell 0)$ are crucial for determining how much they are involved in the quantum dynamics. In Figure S5 we present the populations of each mode for (a) $\mu = 30$ D and (b) $\mu = 72$ D which reflects the evolution in region II and region III respectively. It is clear that in both cases, the amplitudes of the off-resonant modes are significant. In particular, in (a) the coupling is below the critical dipole moment and therefore, the largest amplitude is due to the resonant mode (10). In contrast, in (b) the coupling is larger than at the critical dipole moment, and the off-resonant modes have the largest amplitude.

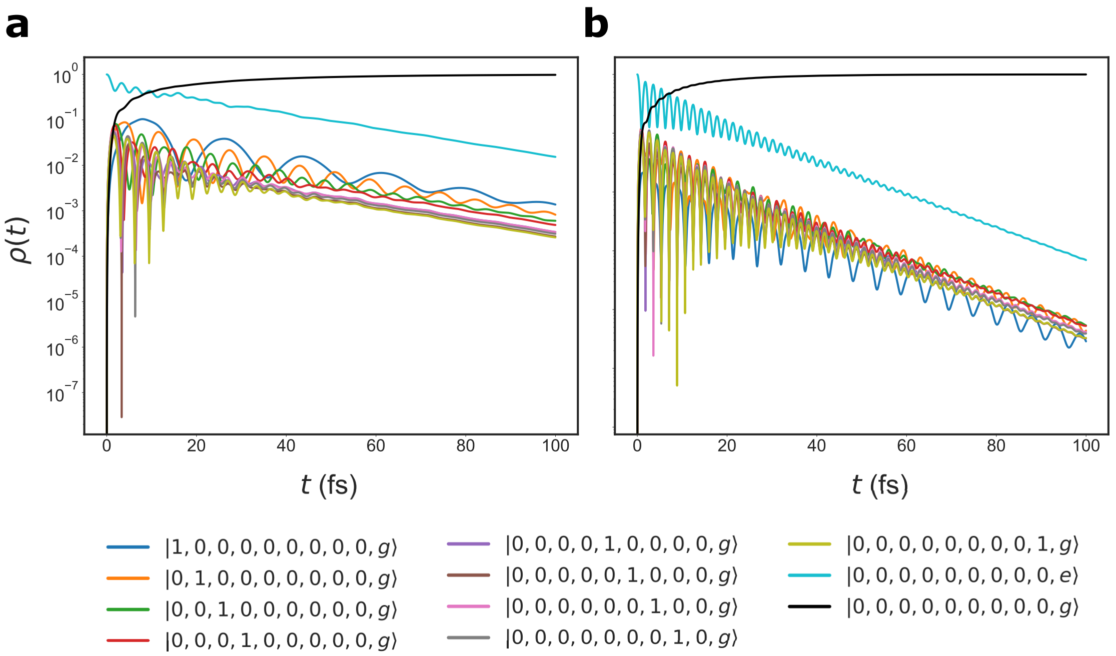

Figure S5: QE and QNM populations as a function of time for (a) dipole moment $\mu = 30$ D and (b) dipole moment $\mu = 72$ D.

To further quantify the quantum dynamics of the modes, we also derive an expression for the mode populations in a system with no loss. From before the coupled equations of motion describing the QE and QNM amplitudes in the interaction picture are given by:

$$i\partial_t c_0 = \sum_{\xi} g_{\xi} c_{\xi} e^{i\Delta_{\xi} t} \tag{S10}$$

$$i\partial_t c_{\xi} = g_{\xi} c_0 e^{-i\Delta_{\xi} t} \tag{S11}$$

where $\Delta_{\xi} = \omega_0 - \omega_{\xi}$ is the detuning between the QE and mode ξ. In addition, in the main text we determined that the QE amplitude takes the form:

$$c_0(t) = \sum_{j=1}^{n+1} \alpha_j e^{i\lambda_j t} \tag{S12}$$

which was calculated through the inverse Laplace Transform of Eq. (9) in the main text. Therefore, we can now substitute Eq. (S12) into Eq. (S11) to also determine the mode amplitudes $c_{\xi}(t)$. Performing this substitution and integrating gives:

$$c_{\xi}(t) = -g_{\xi} \sum_{j=1}^{n+1} \frac{\alpha_j}{x_j} \left(1 - e^{ix_j t}\right) \tag{S13}$$

where $x_j = \lambda_j - \Delta_{\xi}$. Finally, taking the square, and after some simple trigonometric manipulation we derive an expression for the mode populations:

$$|c_{\xi}(t)|^2 = g_{\xi}^2 \sum_{i=1}^{n+1} \sum_{j=1}^{n+1} \frac{\alpha_i \alpha_j}{x_i x_j} \left[1 - \cos\left(\left(\lambda_i - \lambda_j\right) t\right)\right] \tag{S14}$$

which have frequency components at $\lambda_i - \lambda_j$ and we have used $\sum_{j=1}^{n+1} \frac{\alpha_j}{x_j} = 0$. The oscillation frequencies can also be rewritten in the form Ω_i and $\Omega_i - \Omega_j$ by separating the $(n+1)$ th term from both the summations. These frequencies are the same as in the QE populations, as one would expect. For small dipole moments, each mode ξ oscillates at its fundamental frequency Ω_{ξ}. However, as

the dipole moment increases, the mode amplitudes begin to oscillate in phase with one another at the supermode frequency Ω_n. This can be seen in Figure S6 where we plot the number of modes oscillating at the supermode frequency (given by $\mathcal{E}$) as a function of dipole moment.

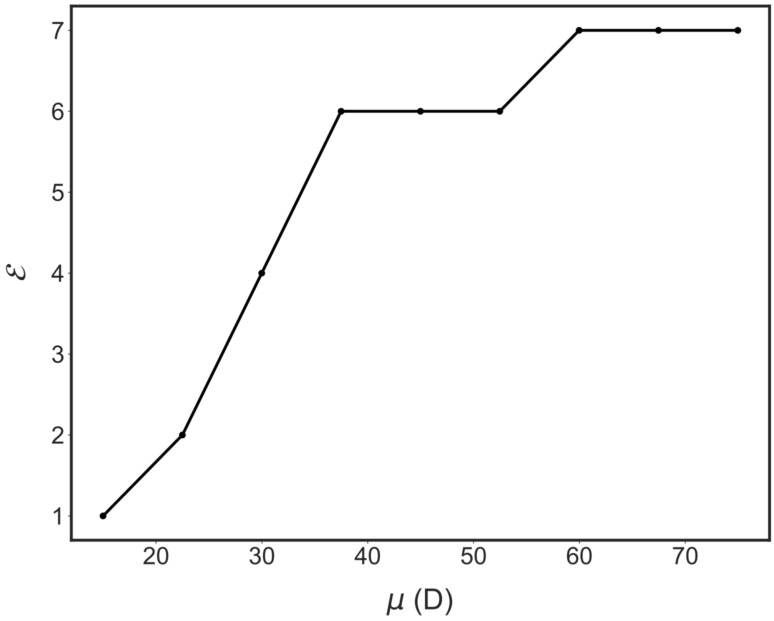

Figure S6: The number of modes that have their largest amplitude oscillation at the supermode frequency ($\mathcal{E}$) as a function of dipole moment (μ). As the dipole moment increases the number of modes oscillating at Ω_n increases.

Collective strong coupling with $m \neq 0$ modes

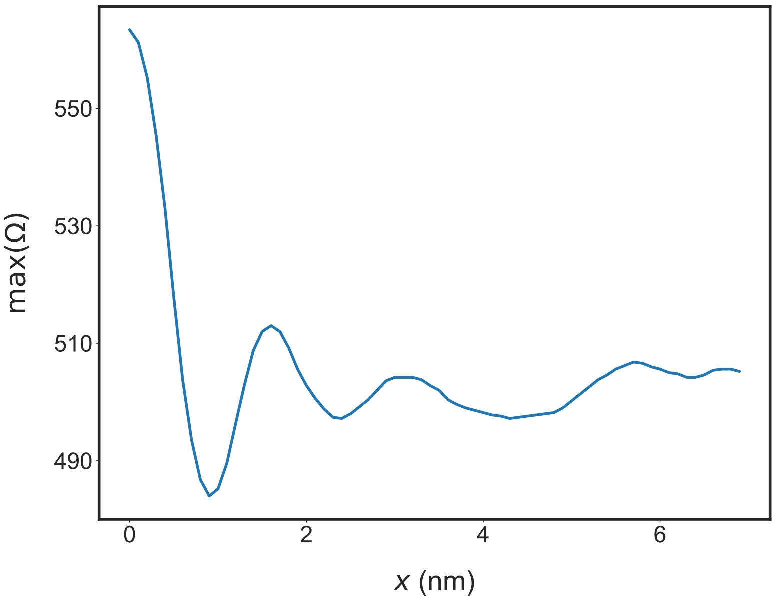

Figure S7: The maximum oscillation frequency max(Ω) as a function of position (x). In general the collective strong coupling regime is maintained due to the large field enhancement of off-resonant modes away from the centre.

In the main text, we show that a single QE at the cavity centre $\mathbf{r}_0 = (0, 0, 0)$ - with dipole moment $\mu = 72$ D - undergoes collective multimode strong coupling with the spherical $\xi = (\ell 0)$ modes. In this case, the quantum dynamics are dominated by a single ultrafast supermode frequency component $|\Omega_n|$. Here, we show that this behaviour is robust to QE displacements, despite the decreasing field strength of the $m = 0$ modes away from the centre.

If the QE is not at the cavity centre, but instead has position $\mathbf{r} = (x, 0, 0)$ it will also couple to the $m \neq 0$ modes that have large field enhancements away from the centre. In Figure S7 we calculate the maximum frequency component in $n(t)$ as a function of displacement x when including all modes up to $\xi = (90)$

which constitutes a total of 103 modes. The ultrafast frequency component $|\Omega_n|$ is maintained for all displacements i.e. we do not see a critical transition back to the resonant mode frequency $|\Omega_1|$.

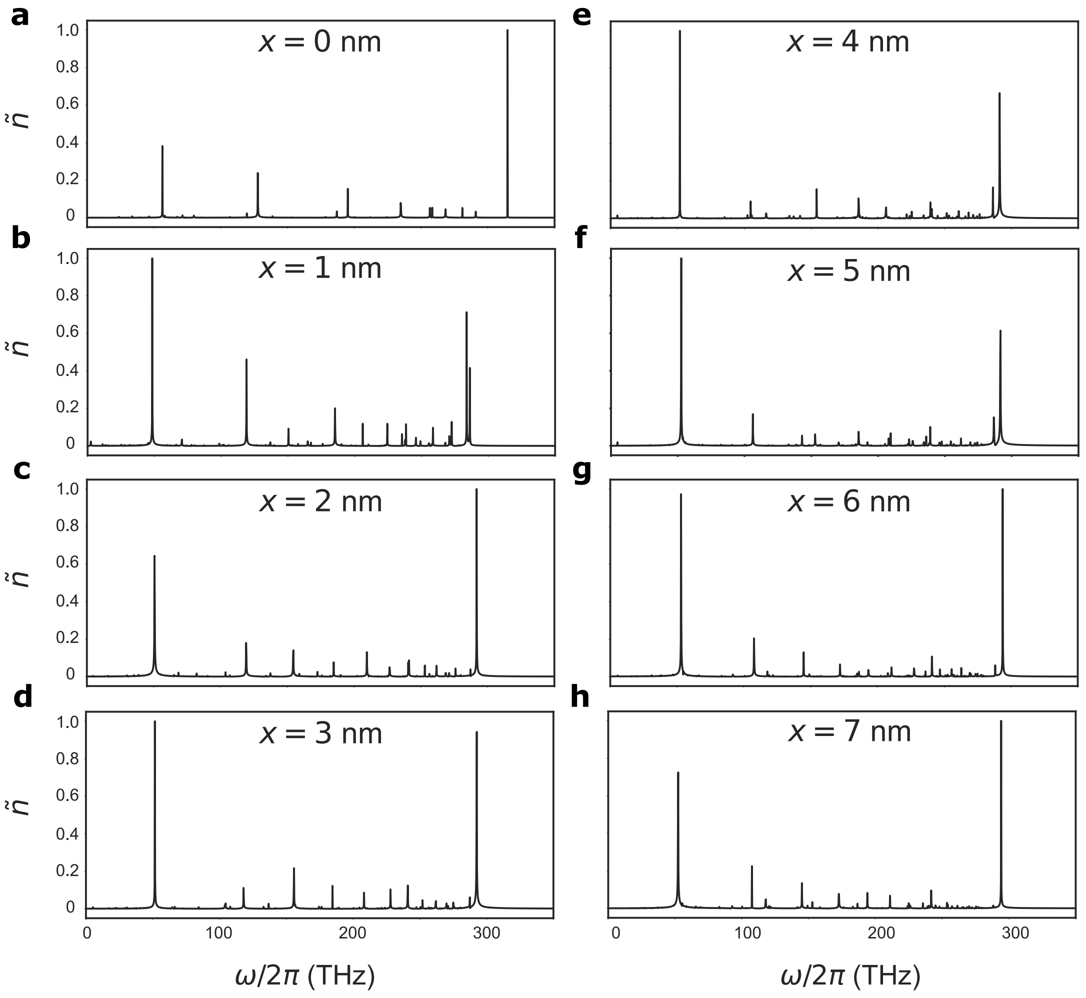

Figure S8: Fourier Transform of the quantum dynamics (without losses) for a QE at position $\mathbf{r}_0 = (x, 0, 0)$ of (a) $x = 0$ (b) $x = 1$ nm (c) $x = 2$ nm (d) $x = 3$ nm (e) $x = 4$ nm (f) $x = 5$ nm (g) $x = 6$ nm (h) $x = 7$ nm. There are always more than one distinct frequency component, and hence, the system remains in the multimode strong coupling regime.

Therefore, the collective multimode strong coupling regime (region III) is maintained for displacements of the QE. To show similarly, that the multimode strong coupling regime (region II) is maintained for displacements of the QE we calculate the frequency components for a smaller dipole moment $\mu = 30$ D for separate displacements x up to $x = 7$ nm. This is shown in Figure S8 (a-h). In general, despite the complex, spatially dependent field distribution of the interacting modes, we always observe more than one distinct oscillation frequency in the quantum dynamics and hence, always remain in the multimode strong coupling regime. Unlike Figure S7 the maximum oscillation frequency changes depending on the position, since the critical dipole moment depends on the coupling strengths of the interacting modes, which in turn depend on the position of the QE.

Dependence of amplitudes on dipole moment

The equation (also stated in the main text) for the evolution of a QEs excited state when interacting with n modes is given by:

$$|c_0(t)|^2 = \sum_{j=1}^{n+1} \alpha_j^2 + 2\alpha_{n+1} \sum_{j=1}^{n} \alpha_j \cos(\Omega_j t) + \sum_{j=1}^{n} \sum_{k\neq j} \alpha_j \alpha_k \cos((\Omega_j - \Omega_k)t) \tag{S15}$$

where the frequencies are given by $\Omega_j = \lambda_j - \lambda_{n+1}$ and the amplitudes by $\alpha_j = \mathrm{Q}^{(n)}(i\lambda_j)/d\mathrm{P}^{(n+1)}/ds|_{i\lambda_j}$ and λ_j are the roots of eq. (S7). In particular, the amplitudes depend on the coupling strengths and detunings of each mode:

$$\begin{aligned} \frac{1}{\alpha_j} &= \left[1 + \sum_{j=1}^{n} \frac{s}{s-\Delta_j} - \sum_{j,k,j\neq k} \frac{g_k^2}{(s-\Delta_j)(s-\Delta_k)}\right]_{\lambda_j} \\ &= \left[1 + \sum_{j=1}^{n} \frac{1}{s-\Delta_j}\left(s - \sum_{k\neq j} \frac{g_k^2}{s-\Delta_k}\right)\right]_{\lambda_j} \end{aligned} \tag{S16}$$

which is evaluated at the root λ_j. Importantly, for small dipole moments the amplitudes are largely independent of the coupling strengths - and instead depend

on the detunings Δ_j and roots λ_j (which are only weakly dependent on g_j). In this region, only the first two terms are important i.e. $\alpha_i \sim [1 + \lambda_i \sum_{j=1}^{n} 1/(\lambda_i - \Delta_j)]^{-1}$ and the largest amplitude is α_1 which occurs for the smallest root i.e. $s = \lambda_1$. For large coupling strengths when $g_k^2 >> |s||s - \Delta_k|$ the g dependent term dominates and the amplitudes will depend on the dipole moment and $\alpha_i \sim [1+\lambda_i \sum_{j=1}^{n} 1/(\lambda_i - \Delta_j) - \sum_{j\neq k} g_k^2/((\lambda_i - \Delta_j)(\lambda_i - \Delta_k))]^{-1}$ In this region, the largest amplitude is α_n which occurs for the largest root i.e. $s = \lambda_n$. Therefore, when the coupling strength is large enough, the largest amplitude peak switches from α_1 to α_n. The crossover to this region occurs at the critical dipole moment.

Full calculations

The oscillation frequencies $|\Omega_j|$ and $|\Omega_j - \Omega_i|$ - which are calculated through the roots of Eq. (S5) - are shown in Figure. S9 in red and green dots respectively for all cutoffs $\ell_{\max}$. They show excellent agreement with the full numerical calculations, which show the Fourier Transform (FT) of the quantum dynamics calculated with no loss ($\kappa_\xi = 0$) through unitary evolution (black lines) and with loss ($\kappa_\xi \neq 0$ obtained from the QNM calculations for each mode) through integration of the Lindblad Master Equation (purple dashed lines). Intermediate steps with $l_{\max} < 9$ illustrate the effect of gradually including more modes coupled to the QE. One would need to include more modes to find the exact supermode frequency , but it doesn't change the qualitative results on how multiple modes impact the quantum dynamics of the system.

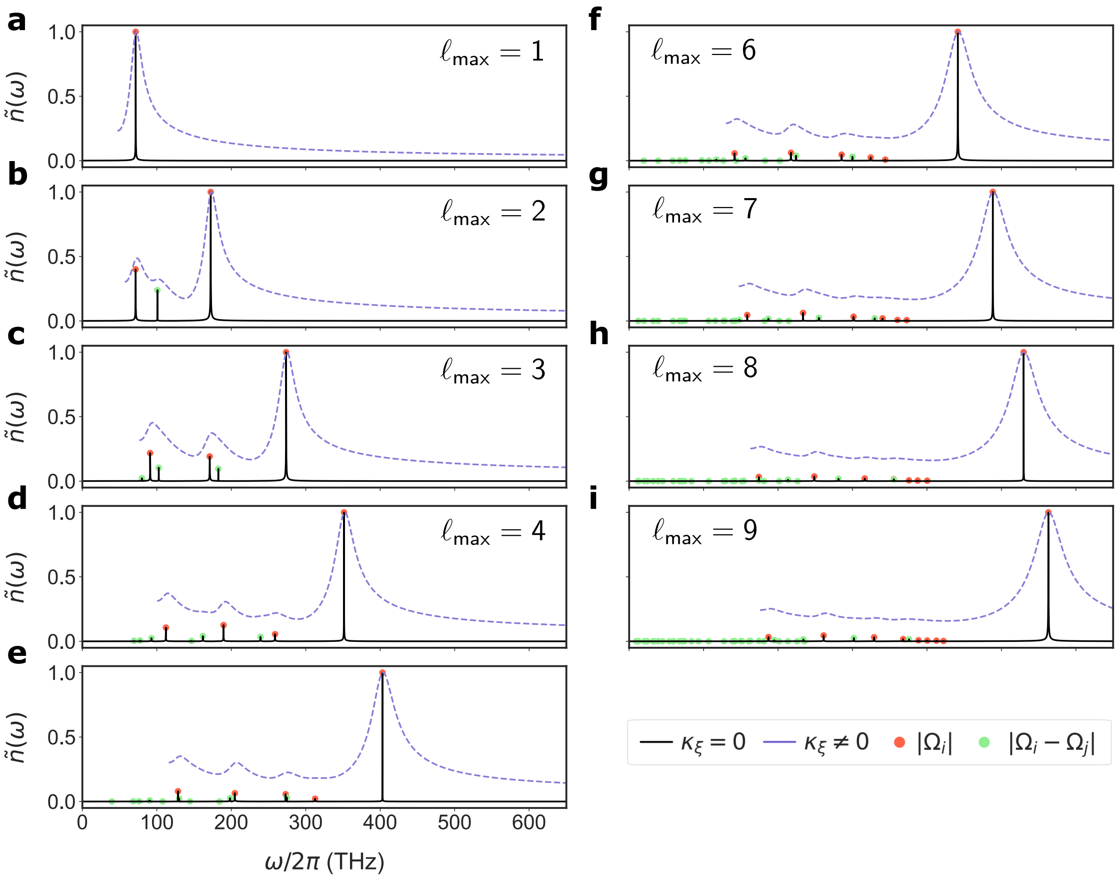

Figure S9: Frequency components in multimode NPoM cavity. The Fourier Transform $\tilde{n}(\omega)$ of the QEs excited state population when including interacting modes up to $\xi = (\ell_{\text{max}}0)$. The solid black lines are numerical results without loss (i.e.$\kappa_\xi = 0$) and the dashed lines with plasmonic loss (i.e. $\kappa_\xi \neq 0$). The red and green dots result from $|\Omega_j|$ and $|\Omega_i - \Omega_j|$ calculated from the roots of Eq. (S5). The sub-figures show the frequencies for when (a) $\ell_{\text{max}} = 1$ (b) $\ell_{\text{max}} = 2$ (c) $\ell_{\text{max}} = 3$ (d) $\ell_{\text{max}} = 4$ (e) $\ell_{\text{max}} = 5$ (f) $\ell_{\text{max}} = 6$ (g) $\ell_{\text{max}} = 7$ (h) $\ell_{\text{max}} = 8$ (i) $\ell_{\text{max}} = 9$ respectively. Note, the spectra with loss are cut to remove the peak at $\omega = 0$ which results from the FT of a damped oscillations.

NPOM and QE resonances

The higher order modes affect the resonances between the NPoM and the QE. This can be observed experimentally using the scattering cross section - which has resonances at the eigenenergies of the Hamiltonian - and is determined by irradiating the system with a weak pump field at frequency ω_{p} and calculating the fraction of light scattered as a function of ω_{p}. The eigenenergies of the Hamiltonian as a function of dipole moment are shown in Figure S10 for both a multimode cavity (solid green lines) and within a single mode approximation (dashed black lines) - the plasmonic mode frequencies ω_{ξ} are also shown for comparison (solid red lines).

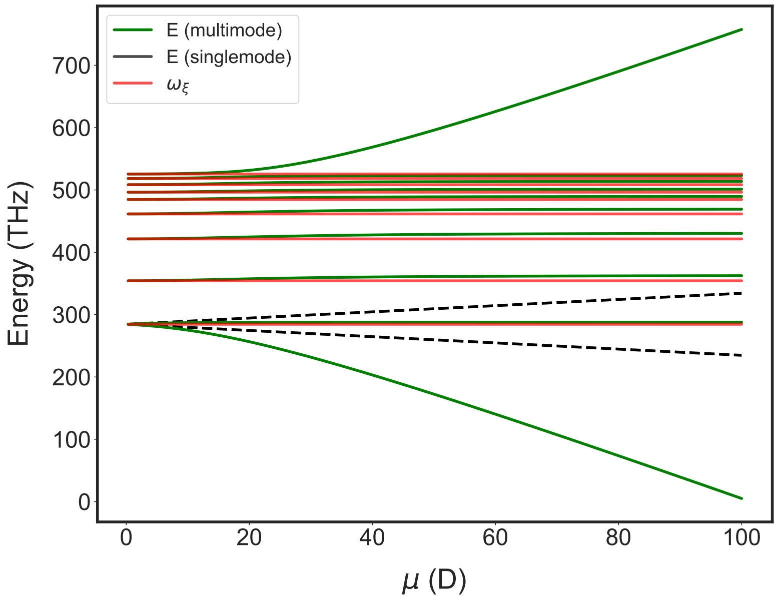

Figure S10: Energy eigenstates as a function of dipole moment for a multimode cavity (solid green lines) a single mode cavity (black dashed lines) and a bare cavity (red lines). The eigenfrequencies correspond to the frequencies measured in the scattering cross section.

In a bare cavity (without the QE) these resonances occur at the mode frequencies ω_ξ. However, when the QE is strongly coupled with just the resonant plasmonic mode (in region I) the (10) mode peak splits into two polariton branches with splitting $|\Omega_1|$ characteristic of single-mode strong coupling. For collective multi-mode strong coupling (region III) the largest splitting will instead occur between the lowest and highest eigenfrequencies, with splitting $|\Omega_n|$. While the relative amplitudes and line-widths of the peaks in the scattering cross section requires a full quantum simulation - similar to that performed for a single mode in [6] - which is beyond the scope of the present work, we expect these to be similar to the linewidths and amplitudes presented in the Fourier Transform of the quantum dynamics in the main text.

Counter-rotating terms

In the main text, we exclude the counter rotating terms (CRTs) which take the form $g_k(\mathbf{r})\left(a_k^\dagger\sigma^\dagger + a_k\sigma\right)$ for coupling with QNM k. The CRTs do not conserve the total number of excitations, as they create or annihilate both the plasmonic mode and QE populations simultaneously, leading to short lived virtual excitations. The effect of the CRTs on the frequency components are shown in Figure S11 for a QE with dipole moment $\mu = 72$ D and resonant frequency $\omega_0 = \omega_{(10)}$. The number of modes included in the interaction is up to $\xi = (60)$ - larger numbers of modes require considerable computational power due the size of the Hilbert space when excitation number is not conserved. The effect of CRTs is to shift the frequency components (due to a Bloch-Siegert shift in the QEs transition frequency). However, this does not remove the collective ultra-fast oscillation.

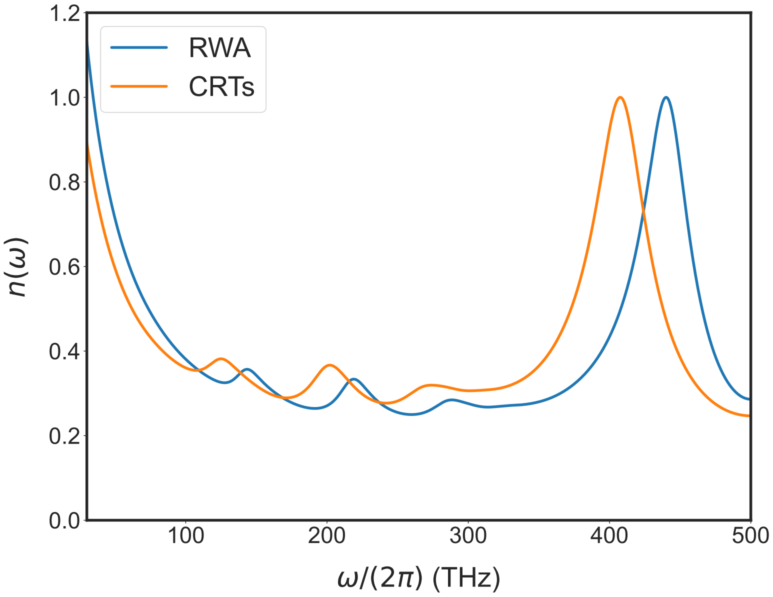

Figure S11: (a) FT including interacting modes up to $\xi = (60)$ within the rotating wave approximation (RWA) and including the counter rotating terms (CRTs)

References

[1] Peter B Johnson and R-WJPrB Christy. Optical constants of the noble metals. *Physical review B*, 6(12):4370, 1972.

[2] Wei Yan, Rémi Faggiani, and Philippe Lalanne. Rigorous modal analysis of plasmonic nanoresonators. *Physical Review B*, 97(20):205422, 2018.

[3] Nuttawut Kongsuwan, Angela Demetriadou, Matthew Horton, Rohit Chikkaraddy, Jeremy J Baumberg, and Ortwin Hess. Plasmonic nanocavity modes: From near-field to far-field radiation. *ACS Photonics*, 7(2):463–471, 2020.

[4] Jeremy J Baumberg, Javier Aizpurua, Maiken H Mikkelsen, and David R Smith. Extreme nanophotonics from ultrathin metallic gaps. *Nature mate-*

rials, 18(7):668–678, 2019.

[5] Stefan A Maier et al. *Plasmonics: fundamentals and applications*, volume 1. Springer, 2007.

[6] Kalun Bedingfield, Benjamin Yuen, and Angela Demetriadou. Subradiant entanglement in plasmonic nanocavities. *arXiv preprint arXiv:2310.06462*, 2023.
